# Comparison of Magnetic Resonance Angiography and Digital Subtraction Angiography for the Assessment of Infrapopliteal Arterial Occlusive Lesions, Based on the TASC II Classification Criteria

**DOI:** 10.3390/diagnostics10110892

**Published:** 2020-10-31

**Authors:** Erik Baubeta Fridh, Karin Ludwigs, Angelica Svalkvist, Manne Andersson, Joakim Nordanstig, Mårten Falkenberg, Åse A. Johnsson

**Affiliations:** 1 Diagnostic Radiology, Department of Translational Medicine, Lund University, Skåne University Hospital, 22185 Lund, Sweden; 2 Department of Radiology, Institute of Clinical Sciences, Sahlgrenska Academy, University of Gothenburg, 41296 Gothenburg, Sweden; karin.ludwigs@vgregion.se (K.L.); marten.falkenberg@vgregion.se (M.F.); ase.johnsson@gu.se (Å.A.J.); 3 Section of Vascular Surgery, Surgical and Ear Clinic, Södra Älvsborg Hospital, 50182 Borås, Sweden; 4 Department of Medical Imaging and Biomedical Engineering, Sahlgrenska University Hospital, 41345 Gothenburg, Sweden; angelica.svalkvist@vgregion.se; 5 Department of Radiation Physics, Institute of Clinical Sciences, Sahlgrenska Academy, University of Gothenburg, 40530 Gothenburg, Sweden; 6 Department of Vascular Surgery, Ryhov County Hospital, 55185 Jönköping, Sweden; manne.andersson@rjl.se; 7 Department of Clinical and Experimental Medicine, Faculty of Health Sciences, Linköping University, 58183 Linköping, Sweden; 8 Department of Vascular Surgery and Institute of Medicine, Department of Molecular and Clinical Medicine, Sahlgrenska University Hospital and Sahlgrenska Academy, University of Gothenburg, 40530 Gothenburg, Sweden; joakim.nordanstig@vgregion.se; 9 Department of Radiology, Region Västra Götaland, Sahlgrenska University Hospital, 41685 Gothenburg, Sweden

**Keywords:** magnetic resonance angiography, MRA, digital subtraction angiography, TASC II, GLASS, chronic limb-threatening ischemia, angiography

## Abstract

This paper aimed to study the agreement and repeatability, both intra- and interobserver, of infrapopliteal lesion assessment with magnetic resonance angiography (MRA), using the TransAtlantic Inter-Society Consensus (TASC) II criteria, with perioperative digital subtraction angiography (DSA) as a reference. Sixty-eight patients with an MRA preceding an endovascular infrapopliteal revascularization were included. Preoperative MRAs and perioperative DSAs were evaluated in random order by three independent observers using the TASC II classification. The results were analyzed using visual grading characteristics (VGC) analysis and Krippendorff’s α. No systematic difference was found between modalities: area under the VGC curve (AUC_VGC_) = 0.48 (*p* = 0.58) or intraobserver; AUC_VGC_ for Observer 1 and 2 respectively, 0.49 (*p* = 0.85) and 0.53 (*p* = 0.52) for MRA compared with 0.54 (*p* = 0.30) and 0.49 (*p* = 0.81) for DSA. Interobserver differences were seen: AUC_VGC_ of 0.63 (*p* < 0.01) for DSA and 0.80 (*p* < 0.01) for MRA. These results were confirmed using Krippendorff’s α for the three observers showing 0.13 (95% confidence interval (CI) −0.07–0.31) for MRA and 0.39 (95% CI 0.23–0.53) for DSA. Poor interobserver agreement was also found in the choice of a target vessel on preoperative MRA: Krippendorff’s α = 0.19 (95% CI 0.01‒0.36). In conclusion, infrapopliteal lesions can be reliably determined on preoperative MRA, but interobserver variability regarding the choice of a target vessel is a major concern that appears to affect the overall TASC II grade.

## 1. Introduction

The prevalence of lower extremity artery disease (LEAD) is increasing worldwide [1,2]. LEAD patients run a high risk of major cardiovascular and lower-limb events, which have a substantial effect on the long-term prognosis and healthcare costs [3]. A subset of patients with more severe symptoms―chronic limb-threatening ischemia (CLTI)―are often eligible for revascularization procedures. A large proportion of CLTI patients have infrapopliteal lesions.

In current practice, the indication for revascularization is based on symptoms and clinical findings, while the revascularization strategy is based on non-invasive preoperative imaging techniques such as magnetic resonance angiography (MRA), computed tomography angiography (CTA) and duplex ultrasonography [4]. In many centers, CTA is the preferred modality for studying lower-limb vessels, because of its non-invasiveness and accuracy but probably mainly due to its high availability [5]. MRA has the advantage of no radiation exposure and also enables the visualization of peripheral arteries with high accuracy [6,7]. Most clinical MRA protocols for the lower extremity are contrast-enhanced with gadolinium-based contrast agents, even if time-of-flight and phase-contrast techniques have also been described. Invasive digital subtraction angiography (DSA) is regarded as the reference method for vascular diagnostic imaging. However, due to its invasiveness and high radiation exposure, DSA is usually only performed during endovascular revascularizations and rarely as a stand-alone diagnostic procedure.

Lesions can be graded according to the TransAtlantic Inter-Society Consensus for the Management of Peripheral Arterial Disease (TASC II) classification, which was introduced in 2007 [8]. The TASC II classification is based on the characteristics of the lesions in terms of anatomic location, the presence of occlusions or stenosis and the longitudinal extent, with the categorization of lesion severity as type A, B, C or D. Several studies have compared different imaging modalities and confirmed the good reproducibility for the aortoiliac and the femoropopliteal arterial segments [9,10]. However, the original TASC II classification did not include the infrapopliteal arteries, which were included in a revision of the classification in 2015 [11].

The aim of this study was to assess agreement and repeatability, both intra- and interobserver, of infrapopliteal lesion assessment with MRA, using the infrapopliteal TASC II lesion criteria and with perioperative DSA as a reference.

## 2. Materials and Methods

### 2.1. Overall Study Design

Preoperative MRA and perioperative DSA examinations were independently evaluated in randomized order by three observers. The observers were blinded to all the clinical patient data. The examinations were assessed according to the infrapopliteal TASC II classification [11].

### 2.2. Patients and Images

All patients aged 50 years and older who underwent an endovascular isolated infrapopliteal revascularization for CLTI between 2008 and 2016 at our tertiary center were identified using the national quality register for vascular surgery (Swedvasc) [12,13]. The age limit was set at 50 years to reduce the risk of including patients with symptoms from causes other than CLTI. The definition of CLTI was a diagnosis of chronic LEAD with ischemic resting pain, ischemic wounds and/or gangrene, by definition, Rutherford 4–6 or Fontaine III or IV [14,15].

Preoperative MRA and DSA images were obtained from the picture archiving and communication system. The contrast-enhanced MRA examinations were performed on 1.5 T magnetic resonance systems using standard clinical protocols. The majority of the examinations (52 of 68) were performed on a whole-body Philips scanner (Achieva; Philips Healthcare, Best, The Netherlands) using a SENSE XL coil between 2008–2014 and a dStream Posterior coil between 2015–2016. The clinical protocol consisted of a three-station subtracted acquisition during the intravenous administration of a gadolinium-based contrast agent (gadoterate meglumine, Dotarem^®^, Guerbet, Roissy CDG, France, or, in some of the earlier examinations, gadobutrol, Gadovist^®^, Bayer Inc., Toronto, ON, Canada) with volumes of 12, 8 and 10 mL for the lower leg, thigh and pelvis respectively, using a 3D T1 weighted sequence and a contrast flow rate of 1 mL/s. The parameters were TR/TE: 4.7 ms/1.5 ms; feet-head coverage: 450 mm; acquired voxel size: 1.4 × 1.4 × 2 mm and reconstructed voxel size: 0.7 × 0.7 × 1 mm. A minority of the examinations (16 of 68) were performed at local hospitals in our region. Ten examinations were performed on a Siemens Symphony (Siemens Healthcare, Erlangen, Germany) and the rest on a Philips Intera. Only static 3D maximum-intensity projections (MIP) and/or dynamic MIP series, depending on the clinical protocol, were presented to the observers. All DSA studies comprised imaging documentation captured during endovascular revascularization procedures.

### 2.3. Image Evaluation

A dedicated study manual, containing standardized image interpretation, was developed to reduce the risk of misinterpretation. For the same reason, an initial consensus meeting was held in which six patients, who were not included in the actual study, were analyzed and discussed.

**Figure 1:**
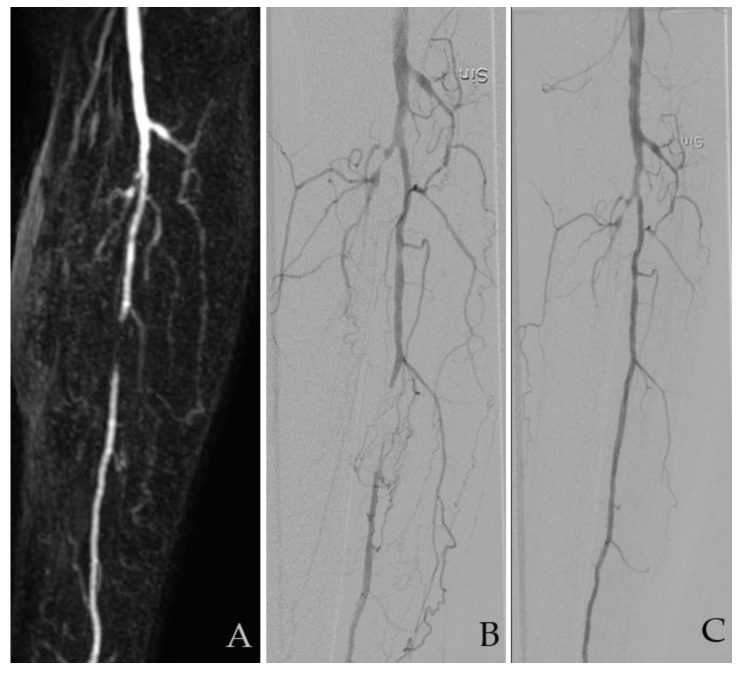
A 68-year smoking patient with type-2 diabetes. He presented with ischemic wounds and gangrene dig 1. Images showing an occlusion in the anterior and posterior tibial artery and a short proximal occlusion in the fibular artery (FA). (**A**) Magnetic resonance angiography (MRA) and (**B**) digital subtraction angiography (DSA) before and (**C**) after revascularization. All four observers graded this as TASC B with FA as a target vessel. Sin: Sinister/left. TASC = TransAtlantic Inter-Society Consensus for the Management of Peripheral Arterial Disease.

**Figure 2:**
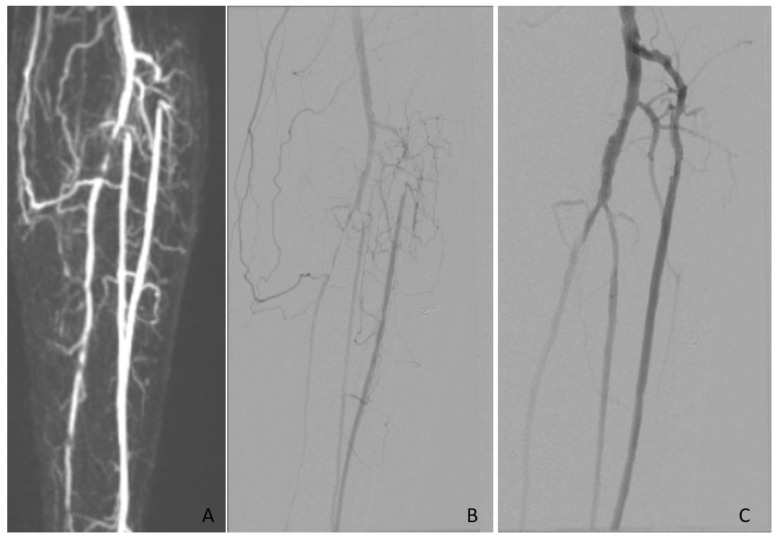
In this patient all three infrapopliteal vessels were affected and revascularized. Images showing short proximal occlusions in the anterior and posterior tibial artery and the fibular artery. (**A**) Magnetic resonance angiography (MRA) and (**B**) digital subtraction angiography (DSA) before and (**C**) after revascularization.

The image evaluations were performed using ViewDEX, which is an in-house-developed image viewer dedicated to research and optimization tasks in medical imaging [16,17,18]. The anonymized images were displayed in a unique random order for each observer, on a high-resolution medical-grade flat panel in a room with ambient low light. Four observers participated in the study. Observer 1 and 2 evaluated the full set of MRAs and DSAs twice each, starting with MRA. Observer 3 evaluated one set of MRAs and Observer 4 evaluated one set of DSAs. As a result, each study was reviewed by three observers for interobserver analysis and twice by two observers for intraobserver analysis. At least one week elapsed between the evaluations of each imaging modality. The readers were interventional radiologists or vascular surgeons with experience in interpreting vascular images ranging from five to twenty-five years. Only patients in whom both the MRA and DSA were assessed as having sufficient diagnostic quality for TASC II classification by both observers were included in the analysis. Examples of the assessed images are presented in Figure 1 and Figure 2.

### 2.4. Statistics

Visual grading characteristics (VGC) analysis [19,20] was used in the statistical evaluation of TASC II classifications when comparing modalities, observers and repeat classifications by the same observer. The analysis results in an area under the curve (AUC_VGC_) that describes the degree of separation between two datasets. A random-reader analysis was employed for comparisons between modalities and for level of confidence, while a fixed-reader analysis was used for inter- and intraobserver comparisons.

To estimate intra- and interobserver agreement, Krippendorff’s α was used [21]. Lower α values correspond to weaker agreement, with greater differences between observers. An α value of 0 corresponds to pure chance, whereas an α value of 1 corresponds to perfect agreement. Cut-off levels for acceptable Krippendorff’s α values depend on the type of agreement that is studied. Commonly, a Krippendorff´s α value of ≥0.67 is regarded as an acceptable agreement. Values above 0.8 are thought to show good agreement between observers.

The statistical software used was SPSS version 24 (IBM Corp., Armonk, NY, USA).

### 2.5. Ethics

Ethical approval was obtained from the ethics committee at the University of Gothenburg (entry nos. 220-17 (26 April 2017) and T1060-17 (4 December 2017)). For this type of study, individual patient consent was not required.

## 3. Results

**Figure 3:**
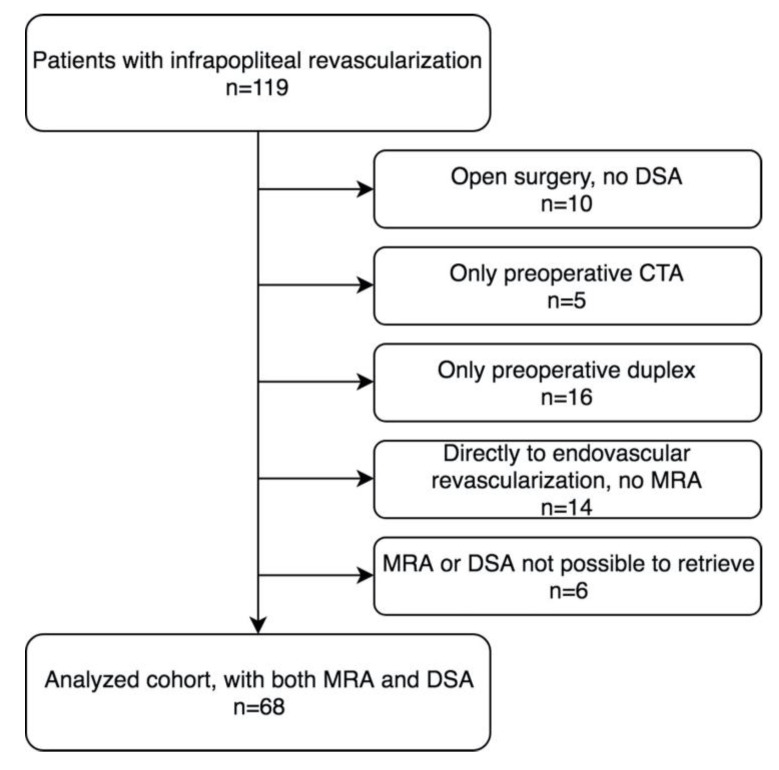
Flow chart showing the selection of the study cohort.

In all, 119 patients with infrapopliteal revascularization were identified. In 68, both preoperative MRA and perioperative DSA images were available and were included in the analysis. A flow chart of the cohort is presented in Figure 3.

Descriptive statistics on the number of examinations with sufficient diagnostic quality and the distribution of assessed TASC II classes are presented in Table 1.

### 3.1. Modality Comparison

In the VGC analysis for modality comparison, only patients in whom all three observers had deemed the diagnostic image quality to be sufficient for TASC II classification of the lower leg were included (*n* = 46). This resulted in a paired data analysis which revealed no statistically significant difference between MRA and DSA in TASC II classifications (AUC_VGC_ = 0.48, *p*-value 0.58).

### 3.2. Assessment of TransAtlantic Inter-Society Consensus for the Management of Peripheral Arterial Disease (TASC) II

In VGC analysis for repeated classification (intraobserver comparison), no statistically significant differences were found for either of the observers. The AUC_VGC_ value for Observer 1 was 0.49 (*p* = 0.85) for MRA (*n* = 61) and 0.54 (*p* = 0.30) for DSA (*n* = 62). The corresponding AUC_VGC_ values for Observer 2 were similar: 0.53 (*p* = 0.52) for MRA (*n* = 49) and 0.49 (*p* = 0.81) for DSA (*n* = 60).

The intraobserver agreement on the TASC grade assignment resulted in Krippendorff’s α values of 0.15 (95% CI −0.30–0.55, *n* = 61) for MRA and 0.49 (95% CI 0.25–0.70, *n* = 61) for DSA for Observer 1. For Observer 2, the corresponding values were 0.58 (95% CI 0.34–0.78, *n* = 46) for MRA and 0.69 (95% CI 0.46–0.88, *n* = 60) for DSA.

Differences were found in the interobserver analysis between Observer 1 and Observer 2 with AUC_VGC_ values of 0.63 (*p* < 0.01) for DSA (*n* = 61) and 0.80 (*p* < 0.01) for MRA (*n* = 51). Similar results were revealed when analyzing interobserver agreement within modalities using Krippendorff’s α. Interobserver α values for the three observers gave 0.13 (95% CI −0.07–0.31) for MRA and 0.39 (95% CI 0.23–0.53) for DSA (Table 2).

### 3.3. Selection of Target Vessel

The intraobserver agreement on choosing the target vessel was fair with Krippendorff’s α values of 0.57 (95% CI 0.35–0.0.76, *n* = 61) for MRA and 0.57 (95% CI 0.35–0.77, *n* = 61) for DSA for Observer 1. For Observer 2, the corresponding values were 0.53 (95% CI 0.25–0.77, *n* = 46) for MRA and 0.60 (95% CI 0.34–0.82, *n* = 60) for DSA.

In contrast, the interobserver agreement regarding the selection of a target vessel was poor (Table 3). For MRA (*n* = 51) and DSA (*n* = 61) agreement between the three observers gave a Krippendorff’s α value of 0.19 (95% CI 0.01–0.36) for MRA and 0.41 (95% CI 0.24–0.56) for DSA. The target vessel, being revascularized peri-operative according to medical charts, was distributed as: tibiofibular trunk *n* = 17 (25%), anterior tibial artery *n* = 19 (27.9%), posterior tibial artery *n* = 11 (16.2%) and fibular artery *n* = 16 (23.5%). In five cases (7.4%) the operators were not able to perform a revascularization procedure. The agreement between the choice of target vessel on preoperative MRA and the target vessel that was eventually revascularized was also poor, with Krippendorff’s α values of −0.02, 0.14 and 0.39 for the individual observers. In 20 of the 68 cases (29.4%), more than one infrapopliteal vessel was eventually revascularized.

## 4. Discussion

There are two main findings in this study. First, no difference in infrapopliteal TASC II classification was found between modalities when the assessment was based on MRA rather than DSA; Second, the interobserver agreement on infrapopliteal TASC II assessment was poor, which might be explained by the poor consensus on choosing the target vessel.

Previous research has shown that MRA gives a correct assessment of arterial infrapopliteal lesions compared with the reference method, DSA [6,7]. The diagnostic properties that were previously compared have been restricted to lesion detection, image quality and signal-to-noise ratio. However, MRA and DSA have not been compared in terms of clinically oriented and internationally established grading systems. The TASC II classification is based on lesion characteristics of importance for the outcome of vascular intervention. Whether the assessment of infrapopliteal TASC II can be accurately made with MRA as compared to DSA has not previously been studied. This study adds important knowledge to this field, since a grading system would not be particularly useful if the assessments between routinely used imaging modalities were inconsistent. In the present study, there were no systematic differences regarding TASC II classification between the two modalities.

However, agreement on TASC II grade was poor between observers, with a numerical level of agreement between observers approaching the likelihood of pure chance. The same poor agreement was found in the selection of a target vessel for an intended revascularization procedure, which the infrapopliteal TASC II classification requires. The low agreement that was observed applied to both modalities. One probable explanation for the poor TASC II agreement might be the requirement to choose a target vessel. This possibly introduces several limitations to clinical and scientific use. Firstly, different observers and operators may choose different target vessels. Secondly, particularly in endovascular procedures, more than one target vessel may be revascularized. In some patients, there is a potential benefit to be gained from revascularizing more than one vessel to achieve wound healing [22]. This was also confirmed in our cohort, as nearly 30% of the performed revascularizations targeted more than one vessel. Thirdly, the choice of a target vessel may also be influenced by the choice of revascularization technique, open or endovascular, potentially hampering comparisons of results between open and endovascular strategies.

In this study, we used the TASC II classification, as this was the main classification available when the study was performed. In 2019, the new Global Anatomic Staging System (GLASS) score was introduced after the image evaluation of this study was completed, and it was therefore not included [23]. However, the GLASS score also requires that a single target vessel is selected for infrapopliteal assessment and hence these results are likely applicable also to this new classification.

The limitations of this study include the retrospective design and the fact that only patients with sufficient image quality for both modalities were included. The strengths include the use of consecutive patients with CLTI from clinical routine healthcare, a structured reviewing process with the ViewDEX software and the random-order image evaluation by blinded observers. Furthermore, we also analyzed the variability of the reference method (DSA) and not only for the investigated index method (MRA).

Since MRA is one of the leading modalities in the preoperative assessment of peripheral vascular lesions, these findings add new knowledge on how to accurately diagnose and predict outcomes in these patients. Further studies need to be conducted to elucidate whether the poor agreement is due to the choice of the target vessel or whether there are other explanations and ways of overcoming these issues.

## 5. Conclusions

Infrapopliteal lesions can be reliably determined with preoperative MRA, but interobserver variability regarding the choice of a target vessel is a major concern which appears to affect the overall TASC II grade.

## Figures and Tables

**Table 1:** Distribution of TASC II classes according to modality and identity of observer. TASC II = TransAtlantic Inter-Society Consensus for the Management of Peripheral Arterial Disease II; MRA = magnetic resonance angiography; DSA = digital subtraction angiography.

	Modality	Examinations Assessed	Good Diagnostic Quality (%)	Assessed TASC II Class, *n* (%)				
Observer 1	MRA	68	62 (91.2)	0 (0.0)	5 (8.1)	11 (17.7)	36 (58.1)	10 (16.1)
Observer 2	MRA	68	52 (76.5)	1 (1.9)	16 (30.8)	25 (48.1)	10 (19.2)	0 (0.0)
Observer 3	MRA	68	64 (94.1)	0 (0.0)	15 (23.4)	25 (39.1)	23 (35.9)	1 (1.6)
Observer 1	DSA	68	62 (91.2)	0 (0.0)	11 (17.7)	14 (22.6)	30 (48.4)	7 (11.3)
Observer 2	DSA	68	62 (91.2)	0 (0.0)	14 (22.6)	28 (45.2)	16 (25.8)	4 (6.4)
Observer 4	DSA	68	65 (95.6)	1 (1.5)	18 (27.7)	32 (49.2)	13 (20.0)	1 (1.5)

**Table 2:** Krippendorff’s alpha (95% CI) for interobserver agreement on assessed infrapopliteal TASC grade.

Infrapopliteal TASC	*n*	α (95% CI)
MRA Observer 1, Observer 2 and Observer 3	51	0.13 (−0.07–0.31)
MRA Observer 1 and Observer 2	51	−0.16 (−0.56–0.2)
MRA Observer 1 and Observer 3	62	0.08 (−0.26–0.40)
MRA Observer 2 and Observer 3	51	0.25 (−0.04–0.52)
DSA Observer 1, Observer 2 and Observer 4	61	0.39 (0.23–0.53)
DSA Observer 1 and Observer 2	61	0.43 (0.18–0.64)
DSA Observer 1 and Observer 4	62	0.25 (−0.02–0.49)
DSA Observer 2 and Observer 4	62	0.45 (0.11–0.72)

**Table 3:** Krippendorff’s alpha (95% CI) interobserver agreement on the target vessel assessed for MRA and versus actually revascularized vessel.

Target Vessel	*n*	α (95% CI)
MRA Observer 1, Observer 2 and Observer 3	51	0.19 (0.01–0.36)
MRA Observer 1 and Observer 2	51	0.12 (−0.13–0.35)
MRA Observer 1 and Observer 3	62	0.26 (−0.00–0.50)
MRA Observer 2 and Observer 3	51	0.13 (−0.22–0.46)
MRA Observer 1—revascularized vessel DSA	57	−0.02 (−0.33–0.26)
MRA Observer 2—revascularized vessel DSA	49	0.39 (0.06–0.67)
MRA Observer 3—revascularized vessel DSA	59	0.14 (−0.20–0.44)
